# Conclusions of the Interallied Surgical Conference

**Published:** 1917-12

**Authors:** 


### SURGICAL

**_Conclusions of the Interallied Surgical Conference._** Third Session, November 1917. Trans. of the Official Stenographic Report.

### I. — Disinfection of Wounds.

Since our last session the disinfection of wounds has passed more and more from the realm of chemical treatment to that of mechanical treatment by surgical means. Primary suture of wounds has taken precedence over secondary suture as a general method of treatment.

*Primary suture*, which we had formerly regarded as exceptional, has now become the method of preference. It has been practiced: as *immediate primary* and *delayed primary* (or early secondary).

*Immediate primary suture* may, it appears, be safely practiced far beyond the time limits we had fixed for light wounds.

*Delayed primary suture* consists in mechanical sterilization of the wound — and in the complete surgical closing of the wound at a later period of from 1 to 4 days after the cleansing operation, and after bacteriological verification.

Among the *organisms* the presence of which is likely to bring about serious complications after the suture, those of first importance are the streptococcus and anaerobic species, especially the spore-bearing variety[1].

### II. — The Value of Bacteriology in War Surgery.

Laboratories annexed to the surgical centers have rendered the greatest service in the matter of surgical technique. Every effort should be made to perfect them, as regards both the personel and the equipment.

At present it is impossible to give any concise bacteriological formula to serve as an indication for the closing of wounds.

1. MM. Castellani and Sacquépée call attention also to the danger from certain other varieties, especially of the Proteus group.

There is need occasionally to call together the bacteriologists from the surgical centers, so that they may discuss the results of their methods and give each other the benefit of their various experiences.

### III. — Gaseous Gangrene : Treatment.

Because of acidosis, in some cases of gas gangrene, an *alkaline* treatment[1] has been instituted. This may be for the purpose of rendering a later operation possible in the case of patients whose condition does not permit of a primary operation; or it may be employed for disintoxication. Encouraging results have been obtained by this treatment. Further attempts should be made in this direction.

Some cases have been treated by various *specific sera* : anti-Perfringens[2], anti-Vibrion septique[3], anti-Bellonensis[4].

These three sera have produced no unfavorable results.

The anti-Perfringens serum, which has been used preventively in several cases, seems to have given encouraging results. As a cure it has been sufficiently successful to warrant further attempts.

The use of anti-Vibrion septique and anti-Bellonensis has given very marked results, by way of cure as well as of prevention, even when administered in very advanced stages of toxic forms of the disease. These results make further tests desirable.

### IV. — Shock.

Since our last session the therapeutics of shock have been modified.

Three causal agents of the state of shock are to be distinguished: hemorrhage. infection or subacute intoxication, and genuine shock.

It is very difficult to distinguish these three conditions. The number of red corpuscles in the veinous blood, if the count is made during the first hour after the wound, taking into consideration the time that has elapsed, makes it possible to detect any serious hemorrhage.

So far, this method has been applicable only in the case of limb

1. Intravenous injection of 500 gm. of the following solution, sterilized in the autoclave : bicarbonate of soda, 50 gm.; distilled water, to 1000.
2. Serum prepared at the Pasteur Institute by M. Veillon.
3. — — MM. Nicelle and Jouau.
4. — Laboratory of the IVth Army (France) by M. Sacquépée.

wounds. In these, however, any marked reduction in the number of red corpuscles is an indication of serious hemorrhage[1].

Under these circumstances *transfusion* has proved successful.

In genuine shock, *warming* of the patient is the essential in any treatment. All beds intended for shock patients should be heating beds.

English surgeons in treating shock make use of *intravenous injections* of a 4 o/o bicarbonate of soda solution in doses of 500 cc. Other saline solutions have so far given no favorable results.

### V. — Artificial Members.

The experience of the past months has confirmed the superiority of the appliances of rigid material, of which the so-called American appliance is the type, in preference to all reinforced leather appliances. They are to be employed after the stump has completed its development.

A temporary appliance, employed as soon as the amputation wound permits, hastens considerably the development of the stump.

### VI. — Surgical Treatment of War Fractures.

The surgical treatment of war fractures has made great progress.

1. Organization.

*a*) Distribution from the rescue station of good immobilization appliances (Thomas splint) and the immediate removal of cases from the rescue stations to the surgical center.

*b*) The increase in the number of specialized surgical centers.

*c*) Perfect uniformity among the different squads, thus permitting absolute unity of treatment at the different stages.

2. Technique of Operation.

Primary reunion of diaphysary fractures has become much more common.

About 51 o/o of diaphysary fractures by shell fragments are treated primarily. In order of frequence these are for wounds of the fore-arm, arm, leg, and thigh (of the last only 20 to 25 o/o are treated primarily).

The following principles seem established: Before all, transform an open war fracture into a closed one, whether by primary suture

1. Less than 4,500,000 during the first 3 hours.
— 4,000,000 — 8 —
— 3,500,000 — 12 —

practiced at once, or after some delay permitting of bacteriological control.

Secondary suture may be performed after chemical desinfection combined with bacteriological control.

3. TREATMENT OF DIAPHYSEAL FRACTURES OF THE THIGH.

Generally speaking, ultimate results of the treatment of thigh fractures have been mediocre.

This fact is due to the extent of such lesions, to the insufficiency of reduction and fixation, and, especially, to the infection of the region of the fracture.

Ultimate results from fractures of the upper and lower thirds of the femur are most defective.

Late complications most frequently noted are: 1. Infections in the form of chronic osteomyelitis; 2. Difformity due to insufficient reduction (shortening, rotation, angulation); 3. Rarely noted defects in consolidation or functional weakness of joints, muscles, or nerves.

Among the deformities, shortening is the most frequent and the only one which cannot always be avoided, for it may result from actual loss of bone substance.

Rotation and angulation are generally due to insufficient surgical care.

Primary sterilization is the first condition to be realized. Defective results are usually due, directly or indirectly, to incomplete sterilization. The delay and difficulty attending the treatment of prolonged infections render fixation much more difficult.

Primary suture of the wound and secondary suture after scar formation, practiced for the purpose of transforming an open fracture into a closed one, may exercise a chief influence upon the ultimate results.

The very frequent stiffness of the knee and that of the hip and foot, whether separate or associated, may be avoided by early exercise.

Muscular adhesion to the callus is the cause of various functional disturbances which may require surgical liberation.

Trophic affections (muscular atrophy, edema, vaso-motary disturbances) play a very important rôle and ought always to be the object of early and persistent attention.

Very pronounced irregularities, with or without osteomyelitis, are amenable to osteotomy. In cases complicated with osteomyelitis, the resection of the callus is indicated.

4. WOUNDS OF THE JOINTS.

At the present time complete closing of joint wounds is universally approved. Such good results have been obtained that now when the excision of contused tissue has involved serious loss of subcutaneous substance, thus rendering *direct* closing impossible, this loss may be made good by cutaneous autoplasty. Bony cavities in some cases may be filled with muscular flaps.

The aseptic progress of bone wounds of the joints, when they have been operated upon within ten or twelve hours after the injury, makes possible a maximum of surgical conservation.

Epiphyseal fractures or epiphyso-metaphyseal fragmentation (trabecular fractures, incomplete perforations, etc.) are amenable at present to cleansing and curetting, followed by primary closing of the joint.

Epiphyseal and partial or T-shaped epiphyso-metaphyseal fractures, are amenable, according to the nature of the case, to arthrotomy with reduction, or to arthrotomy with a conservative seque strectomy (partial atypical resection), followed by primary suture.

Primary resection is now only occasionally indicated :

*a*) In case of extensively comminuted fractures of the joints. Even in these cases, the indications are more urgent when the hip or shoulder is involved than when it is a question of the knee, the elbow, or the ankle.

*b*) In questionable cases, the practice of primary resection with a view to obtaining a better functional result, should give way to conservative methods (arthrotomy with conservative sequestrectomy and partial atypical resection).

Defective functional results are amenable to late orthopedic resection, which is usually more effective than primary resection.

Amputation is indicated in cases of injury involving the destruction of the principal arterial trunk.

Immediate active exercise appears to be the indispensable complement of surgical treatment, and has given much better result than immobilization (Willems).

Resection is still desirable in cases of infection of joint fractures.

5. TREATMENT OF CHRONIC OSTEOMYELITIS.

Bone infection is brought about in the same way as infection in other types of war wounds.

It is promoted in two ways.

*a*) Directly through medullar and compact tissue.

*b*) Indirectly along fissures.

Penetration through the bony substance is generally slow and limited in extent.

Among the first bacteria to invade are to be noted : streptococci, staphylococci, enterococci, and,' less frequently, the anaerobes.

In the flora of subacute or chronic bone suppuration, the following organisms are most frequently found : streptococci, staphylococci, enterococci, and pneumobacilli.

The treament for chronic osteomyelitis is chiefly preventive. It consists simply in the proper treament of the fracture.

(Reference is made to the subject of *Fractures* as presented in the conclusions adopted at this and the previous session of the Inter-allied Surgical Conference. For the latter, see the *Medical Bulletin* for November (No. 1), p. 14).

The treatment of established osteomyelitis is at present purely surgical. It should be practiced as early as possible, and should consist principally in :

*a*) Generous opening of the region.

*b*) Minute search for, and removal of, all sequestra and foreign bodies.

*c*) The leveling of all cavities.

After this procedure there are two possible courses open :

*a*) Either the immediate closing of the wound with autoplasty of the gap in the bone by means of flaps ;

*b*) Or chemical sterilization of the wound followed by secondary autoplasty and suture. This last method is the one usually adopted.

### VII. — Chest Wounds.

Surgical treatment of chest wounds tends clearly towards active surgery.

*a*. In case of dangerous hemorrhage, an open thorax is always operated.

*b*. Primary extraction of intra-pulmonary projectiles is more frequently practiced.

Treatment of the parietal wound consists in excision and removal of all foreign matter, whether bone or metal, lodged in the lung, cleansing of the wound, and immediate primary closing of the thorax Good results thus obtained clearly justify this method of surgery.

*c*. Infected hemothorax is treated by very early drainage and progressive disinfection and secondary closing of the thorax.

*d*. Chronic purulent pleurisy is amenable to progressive sterilization, accompanied, if necessary, by the opening of the pleuro-pulmonary casing by several incisions.

### VIII. — Wounds of the Abdomen and Thorax.

In wounds of the abdomen and thorax, the wound of the diaphragm constitutes the specific factor of the lesion; the wounds of the organs of the abdomen and thorax present no special anatomical interest.

Hernia of the abdominal organs is difficult to recognize clinically. Radioscopic examination reveals the displacement of the heart to the right, and so helps in diagnosis so far as the left side is concerned.

Indications for operation are absolute except in the case of a very small projectile in the upper part of the abdomen, especially at the right. The perforation of the diaphragm in itself necessitates suture.

Approach by way of the pleura affords especial advantage for exploration of the lesions and also for treating the pleural cavity or for suturing the diaphragm. It permits also the treatment of lesions in the abdominal organs, beneath the diaphragm, whether there is hernia or not.

A separate laparotomy may sometimes be necessary.

The opening of the thorax and abdomen by a single incision permits generous treament of both chest and abdominal lesions.

### IX. — Brain Wounds.

1. Surgery.

Brain wounds are disinfected mechanically by trephination, cleansing of the cerebral substance with warm water, and the extraction of easily accessible projectiles.

Bacteriological control, in this as in all wounds, will furnish indication for complete closing. In case foreign bodies may not be extracted, primary closing, whether immediate or delayed, ought to be practiced with extreme prudence.

Very great care must be exercised if grafts are attempted.

2. Secondary and Late Complications in Brain Wounds.

All documentary evidence available[1] indicates that although secondary complications in cerebral wounds are comparatively

1. Out of 6,664 cases which had previously undergone trepaning at the hospitals of the neurological regional centers of France, there were recorded 676 cases of partial or general epileptic crises (or 10.14 o/o), 94 cases of late cerebral abcess (or 1.41 o/o), 32 cases of late meningitis (or 0.48 o/o), and 54 cases of late cerebral hernia (or 0.81 o/o). There were 83 deaths (or 1.24 o/o) but only 1 sudden death.

frequent, late infectious complications are much less common than was supposed.

Organic disturbances resulting from wounds of the brain (hemiplegia, monoplegia, aphasia, visual disorders, etc.) frequently improve. The treatment is properly neurological.

Late epileptic seizures of the Jacksonian variety may benefit by surgical treatment for the removal of the cause of pressure, of foreign bodies, and of splinters. It is useless to operate for one or two isolated seizures of Jacksonian epilepsy, for these may be due to an attack of encephalitis which may be cured, and which would not be affected by operation.

Epileptic seizures other than those brought on by foreign bodies or splinters, do not call for fresh surgical treatment. Lumbar punctures, controlled by a manometer, may prove beneficial when there is hypertension of the cerebro-spinal fluid.

Late cerebral abcess, which should be carefully diagnosed and differentiated from non-purulent encephalitis, may be operated after precise localization by the surgeon and neurologist. After an exploratory puncture, such as will not destroy the protective coverings, the abcess should be opened and its sterilization followed bacteriologically.

Late localized meningitis, encysted meningeal abcesses, should be operated.

The present treatment of generalized meningitis is usually of little avail. Repeated lumbar punctures appear to be the most rational.

Cerebral hernias with abcess should be operated and the abcess drained.

It seems best to abstain from lumbar punctures in the primary acute, febrile stage of certain cerebral hernias, thus avoiding the possible diffusion of a local infection. When the initial infectious phenomena have been reduced, lumbar punctures, for the purpose of relieving intracranial hypertension, may help to lessen the hernias.

Resection of hernias is permissible only in case of local necrosis or meningocele.

Foreign bodies in the brain, causing attacks of encephalitis, epileptic seizure, or abcesses, should be removed. It seems best not to disturb those that cause no apparent disorder.

Cranioplasty, judging from all available reports, appears desirable for esthetic reasons in case of frontal cavities. For curative purposes, however, it is justifiable only when the disorder is occasioned exclusively by the extent of the loss of bone substance.

As a preventive measure, the danger of subsequent injury to the brain in the region of the gap may justify the operation. In any case, it must be carefully established that no counter-indications exist; such as, nervous disorders, chemical or cytological change in the cerebro-spinal fluid, and papillar stasis.

Early therapeutic procedure, methodical disinfection, and primary sterilization of the intra-cerebral seat of injury, are the best preventive methods against secondary or late infectious complications.

### X. — Treatment of Nerve Wounds.

Treatment of nerves in war wounds has, up to the present, produced rather poor ultimate results.

In cases of partial section the percentage of good results is much higher than in those of complete section.

Poor results are due in large measure to late treatment.

Treatment ought never to be applied through a suppurating medium.

The three principal causes for failure in late treatment of the nerves are :

*a*) Sclerosis of the peripheral end, increasing with delay.

*b*) Too great separation of the ends.

*c*) Intensity and duration of suppuration.

Retraction of the tendons, ankylosis of the joints, ischemic muscular sclerosis, all tend to lessen considerably the functional value of the result obtained by treatment. From the start a proper corrective attitude should be given to the joints.

Primary nerve suture, made possible by the present methods of wound disinfection, is likely to increase materially the frequency, the rapidity, and the extent of functional recovery.

Even in case of failure, primary suture fixes the nerve ends in an anatomical position which greatly facilitates a new operation.

Functional recovery is slow, requiring months and even years. This fact must be taken into account in judging injuries.

